# Qualitative Study of Emergency Medicine Residents’ Perspectives of Trauma Leadership Development

**DOI:** 10.5811/westjem.60098

**Published:** 2023-12-20

**Authors:** Antionette McFarlane, Sarah M. Brolliar, Elizabeth D. Rosenman, Joshua A. Strauss, James A. Grand, Rosemarie Fernandez

**Affiliations:** * University of Florida, Department of Emergency Medicine, Gainesville, Florida; † University of Washington, Department of Emergency Medicine, Seattle, Washington; ‡ University of Maryland, Department of Psychology, College Park, Maryland

## Abstract

**Background:**

Trauma team leadership is a core skill for the practice of emergency medicine (EM). In this study our goal was to explore EM residents’ perception of their trauma leadership skill development through formal and informal processes and to understand factors that may impact the development and implementation of trauma leadership skills.

**Methods:**

Using qualitative semi-structured interviews, we explored the leadership experiences of 10 EM residents ranging from second to fourth postgraduate year. Interviews were conducted between July 26–October 31, 2019 and were audio-recorded, transcribed, and de-identified. We analyzed data using qualitative content analysis.

**Results:**

Residents discussed three main themes: 1) sources of leadership development; 2) challenges with simultaneously assuming a dual leader-learner role; and 3) contextual factors that impact their ability to assume the leadership role, including the professional hierarchy in the clinical environment, limitations in the physical environment, and gender bias.

**Conclusion:**

This study describes the complex factors and experiences that contribute to the development and implementation of trauma team leadership skills in EM residents. This includes three primary sources of leadership development, the dual role of leader and learner, and various contextual factors. Research is needed to understand how these factors and experiences can be leveraged or mitigated to improve resident leadership training outcomes.

Population Health Research Capsule What do we already know about this issue?

*Trauma team leadership is critical to patient care and an important part of physician development.*

What was the research question?

*Our goal was to identify how residents perceive their development of trauma leadership skills through formal and informal processes.*

What was the major finding of the study?

*This study reveals the importance of individual and team factors in the development of trauma team leadership skills.*

How does this improve population health?

*Understanding factors that influence trauma leadership development could help guide training and educational programming for junior physicians.*

## INTRODUCTION

Leadership impacts the quality of patient care during trauma resuscitations.^1^ Trauma team leaders coordinate care, manage and set priorities, and organize resources.^2^ Such skills are critical under the dynamic, time-pressured conditions present during medical and trauma patient resuscitations. Failure to establish leadership in teams leads to suboptimal teamwork and threatens patient safety.^3^ The medical education literature describes multiple training programs focused on developing trauma team leadership skills.^4^ Research suggests that teaching residents to lead in high-stress environments is critical^5^ ^,^ ^6^; however, there remain gaps and challenges associated with developing critical leadership skills. Currently it is not known how residents experience trauma team leadership, nor is it well understood what factors may contribute to the development of leadership skills. Understanding these experiences and factors can help educators facilitate and optimize learning.

We conducted a qualitative study of emergency medicine (EM) residents with the objective of identifying how residents perceive their development of trauma leadership skills through formal and informal processes and what factors in the clinical environment might support or inhibit development and reinforcement of trauma team leadership skills. This work will help address an important knowledge gap and inform future leadership development efforts.

## METHODS

### Study Design

In this study we used an exploratory qualitative approach to examine EM residents’ perception of trauma leadership skill development and understand factors that may impact the development and reinforcement of team leadership skills. We conducted semi-structured interviews between July 26–October 31, 2019. The University of Washington Institutional Review Board and the University of Florida Institutional Review Board approved this study as no greater than minimal risk. We used the Consolidated Criteria for Reporting Qualitative Research Checklist (COREQ) in preparation of this manuscript.

### Setting and Study Population

We recruited a purposive sample of EM residents in their second through fourth years of postgraduate education because of their knowledge and experience as team leaders during trauma resuscitations. Participants were recruited from the University of Washington EM residency program. To recruit participants, we sent an email to the EM residency listserv containing the study description and a brief survey to elicit interest in study. The study coordinator (SMB) also conducted brief in-person informational sessions about the study during monthly EM residency conferences. Eleven EM residents were selected to participate in the study, with one resident declining due to a family emergency. Participation was voluntary. Recruitment concluded when data saturation^7^ was reached, and no new insights were identified in the interview data. All participants were given a $25 gift card as compensation.

### Interview Guide and Data Collection

Using an iterative process, we developed an interview guide to elicit participants’ perspectives on trauma team leadership development. Question development was guided by a review of leadership and training literature.^4^ Questions were pilot-tested with an EM resident and revised using feedback and suggestions from the resident and the research team. The interview guide underwent a second round of testing prior to implementation (See Supplemental Material [Interview Questions].) We did not include data from pilot-testing in the final results.

We collected data using semi-structured, face-to-face interviews that included probing questions and follow-up questions to gain more in-depth explanations and clarification. Participants answered questions regarding source of leadership skills, implementation of leadership skills, formal leadership training, and the dissemination of team leadership skills. All interviews were conducted in a private location with only the researcher and participant present. Interviews were conducted by SMB, who had prior experience conducting qualitative interviews and had no prior relationship with participants. Interviews ranged from 33–85 minutes, with a mean length of 43 minutes. Demographic information was collected through a secure online survey administered through REDCap, an electronic data capture tool hosted at the University of Washington. Interviews were audio-recorded, and recordings were transcribed verbatim by a professional service, followed by redactions of identifying information by the original interviewer. Transcripts were not returned to participants for review.

### Data Analysis

Due to the limited research on leadership skill development, we used qualitative content analysis for data analysis.^8^ Drawing on this method, researchers first immersed themselves in the data. Three members of the research team (AM, SMB, and JS) with previous qualitative experience served as coders and read through each of the transcripts several times. Then, using open coding^8^ ^,^ ^9^ all three coders independently reviewed one of the interview transcripts and developed a set of initial codes to represent ideas and phrases revealed in the data. The coders met weekly to compare preliminary codes, discuss differences, and expand or collapse the codes as needed.

To ensure reliability of the codes, codes were further reviewed by the entire research team during bi-weekly meetings. These codes were used to construct a codebook, which included code names, definitions, and exemplar quotes. The codebook was then applied to two additional transcripts by all three coders, and the codes were refined through discussions with the entire research team. Using the finalized codebook, each remaining transcript was then coded by two of the coders. Coders met to review transcripts and discuss any disagreements until consensus was reached. Codes were analyzed across transcripts to identify categories and major themes, which were reviewed and modified by the larger research team. Coders summarized major themes and finalized results presented in this paper. Dedoose version 9.0.47^10^ (SocioCultural Research Consultants, LLC, Manhattan Beach, CA) was used to assist in coding and organizing data. Participants did not provide feedback on the findings.

## RESULTS

We conducted a total of 10 interviews. Participant demographics are described in the Table. This paper presents three primary themes that emerged from the data: 1) sources of leadership development; 2) dual learner-leader role; and 3) contextual factors impacting leadership. Supplemental Material (Central Themes and Exemplar Quotes) summarizes primary themes, subthemes, and representative quotations.

**Table:** Participant demographics (N = 10).

Demographic	Participants (n = 10)
Age, year; mean (SD)	30(3)
Male, n(%)	5(50)
Race, n(%) ^a^	
American Indian or Alaskan Native	0(0)
Black	0(0)
Native Hawaiian or other Pacific Islander	1(10)
Asian	2(20)
White	9(90)
Other	0(0)
Ethnicity n(%)	
Hispanic or Latino	0(0)
Not Hispanic or Latino	10(100)
Residency year, n(%)	
PGY 2	3(30)
PGY 3	3(30)
PGY 4	4(40)

a Participants were able to select more than one racial category.

*PGY*, postgraduate year.

### Sources of Leadership Development

Drawing on life and work experiences, residents discussed the development of their leadership skills. Residents cited three primary sources: 1) observing senior residents and attendings; 2) supervised leadership practice; and (3) prior life experiences.

The observation of senior residents and attendings was cited as important to participants’ leadership development. They noted that much of their time as junior residents was dedicated to observing others in the trauma leader role. The opportunity to observe senior residents and attendings provided exposure to multiple leadership styles that could inform their own leadership approach. One participant shared the following:

“Two in particular, senior residents when I was an intern that I watched, that I took a lot of learning points from … like how they manage things. And I think what was helpful about those two senior residents in particular, was that I thought their leadership styles were something similar to what I wanted to emulate.” (Participant 1)

Residents also pointed out that these opportunities diminished as they progressed in residency due to time constraints, scheduling factors (eg, not working at the same time as other senior residents), and a shift from being a team member to being the team leader.

Participants also emphasized the importance of closely supervised leadership opportunities in shaping their approach to leadership development, specifically, “low-stakes” supervised clinical practice. Junior residents were often encouraged by senior residents to take the leadership role in lower acuity trauma resuscitations, which provided an opportunity for them to practice their leadership responsibilities before applying them in a higher acuity, more complex situation. These opportunities were not a formalized process but rather depended on the residents’ ability to free themselves from other clinical responsibilities and on their relationship with their senior resident.

In addition to on-the-job training, multiple residents described prior life experiences (eg, academic experience, sports team participation, etc) that provided a foundation of leadership skills and supported their role as trauma team leader. Participants described how these experiences helped to develop communication and team management skills, in addition to preparing them to acclimate to new or stressful environments. One resident stated,

“I mean I guess leadership roles in your past life, like I was president of the [sports club] at my college and so had experience standing up in front of a group of people and like guiding things…there’s a lot of leadership required there …” (Participant 9)

For a few participants, past experiences served to build their *confidence* as a team leader. One resident shared,

“(my past experience impacts) …my own personal confidence in telling other people what to do. Or like asking for help if I don’t know what to do without feeling weird about it, I think more comes from like longer history of just being in those positions, leadership positions, kind of working with other people, teams, directing people, coaching.” (Participant 11)

### Dual Learner-Leader Role

Participants noted that the role of trauma team leader requires residents to simultaneously assume the role of a leader and a learner. As the leader of the resuscitation, the resident is seen as the “central figure” of the resuscitation. Participants indicated that trauma team leaders are responsible for information-sharing (collecting, synthesizing, and disseminating information) and role designation (assigning tasks to team members), as well as conducting the primary trauma survey. Participants shared that they established themselves as the team leader through their speech and their physical location in the room. They also exuded confidence by communicating frequently or by being “vocal,” and in some instances by being “loud,” when instructing the team.

Participants also acknowledged that they were still developing their medical knowledge and learning to manage the trauma team. Residents were not expected to have the expertise of their senior colleagues, and the senior residents were expected to provide supporting functions such as offering medical prompts (“code-whispering”) or coaching. As a resident shared,

“Well, you have your ED attending in there who will kind of bring things up that you may be missing…there’s a surgery senior, there’s a surgery fellow, there’s a surgery attending … who are not going to allow you to kind of miss things. And so, while you should strive to be the person who’s putting together all the plans, there’s also a lot of people who will feed you things that you may have missed.” (Participant 5)

Participants noted that embodying roles of both leader and learner may result in conflict. Specifically, residents suggested that their role as a learner may have impacted their success as trauma team leaders. As early learners, residents indicated that they were still developing their clinical knowledge and this may have overshadowed their focus on leadership responsibilities. A resident shared,

“But it is really difficult, I think, as an early learner, especially as a second-year resident to be able to handle the mob that shows up and try to do good patient care when you’re still trying to learn the clinical medicine.” (Participant 1)

Residents perceived that the trauma leader’s medical knowledge is necessary for effective leadership. As learners, residents are still questioning their medical knowledge and may not feel comfortable providing directions to team members. Additionally, residents’ lack of experience leading trauma resuscitations shaped their confidence in their leadership abilities. A resident stated,

“The other challenge, especially earlier on in training, is if you just don’t have quite as much knowledge; so it’s much easier to be in that role when you feel confident in the plan because you’ve done it a bunch of times and you kind of know what’s supposed to happen next. But if you’re still not quite totally sure about what is the right thing to do next, it becomes very difficult. I think, to kind of be in that more directive role…” (Participant 11)

As learners, residents acknowledged that lack of experience leading trauma resuscitations and lack of medical knowledge directly affected their level of confidence and hindered their ability to lead. Further, a lack of knowledge undermines team members’ confidence in the leader. Residents indicated that if team members thought the leader lacked confidence, a senior colleague could step in and assume leadership of the resuscitation.

### Contextual Factors Impacting Leadership

Participants acknowledged that their leadership could have been impacted by numerous contextual factors including the professional hierarchy, the resident’s gender, and physical workspace limitations. Several of the residents recognized how the professional hierarchy impacts their ability to lead a resuscitation. Although the resident is presumed to be the leader of the resuscitation, they are supervised by senior colleagues with more experience. Participants suggested that some supervision may be an impediment to leadership and deprive them of the opportunity to develop clinical and leadership skills. One resident revealed,

“So for me I find it to be, it’s hard for me to feel like I’m the person in charge when there’s a lot of more senior people in the room that kind of want to butt in and make the decisions.” (Participant 3)

When this occurred, residents shared that it was difficult to challenge the assertions of a senior colleague when team members were more likely to listen to the senior colleague. As a result, residents may adopt a more passive approach to leadership, where they are not assigning roles and tasks, but rather they are receiving instructions from their senior colleagues who ultimately have more authority and experience.

Participants also discussed the impact of gender on their leadership efficacy. Residents mentioned that female trauma team leaders may face additional obstacles while leading. Female residents attributed these obstacles to their physical and vocal attributes such as stature or tone of voice. As one female resident shared,

“You get the assumption that you are not the trauma doc running the resuscitation. Someone told me, ‘I’m getting sick of being handed the oxygen when people walk in the room or being handed things like, ‘Hey, can you throw this away,’ when I need to really be focused on things.” And I was like, ‘Wow, that hasn’t happened to me.’ And then yesterday I got handed the oxygen.” (Participant 8)

Participants of both genders suggested that male residents are assumed to be the leader due to their physical size or their ability to be “loud,” while female trauma team leaders must work to establish themselves as the leader. The assumption that the female resident is not the leader of the resuscitation reinforces a gendered hierarchy where men are viewed as the “leader,” and women are assumed to be in a supporting role.

Additionally, participants often discussed difficulties in leading the team due to limitations of the physical workspace and crowding. Smaller workspaces may obstruct trauma team leaders’ ability to perform procedural tasks. Additionally, an increased number of observers and team members can lead to environmental noise and professional silos. Limited space in the trauma room and number of staff in the physical workspace may critically restrict the ability of the trauma team leader to perform their responsibilities.

## DISCUSSION

Residents identified three primary sources that inform the development of leadership skills: 1) observation of senior residents and attendings during clinical care; 2) supervised leadership practice; and 3) prior life experiences. Interestingly, residents did not highlight formal leadership training delivered within the medical education curriculum, either as medical students or within residency. This may reflect the relative value residents place on various learning modalities and educational resources. These findings are in line with those of Quon et al (2022), which reflects the importance of informal curriculum in shaping the leadership development of EM residents.

Real-world experiences and practice may overshadow more passive learning experiences, such as lectures and online modules. In a prior qualitative review, EM residents reported employing different learning strategies for clinical skills vs leadership skills.^11^ Simulation is frequently lauded as a modality for immersive, hands-on training without compromising patient safety.^12^ ^,^ ^13^ While several residents mentioned simulation-based education, it was not a prominent theme relative to clinical exposure. This is supported by previous work indicating that simulation does not replicate the stress, anxiety, and other challenges encountered during actual clinical care.^14^

Understanding current sources of leadership development will help educators develop more effective leadership training programs. The importance of a needs assessment is highlighted in the training development literature. However, needs assessments in medical education generally evaluate gaps in training or knowledge and are not focused on the environmental, cultural, and organizational factors that directly impact learning.^15^ This can result in failure to appreciate “hidden” educational opportunities and challenges. This study emphasizes the important role that other residents, particularly senior residents, play in shaping junior residents as they develop leadership skills. While the process of graduated responsibility from observation (via team membership) to low-acuity practice to team leadership is not novel, participants highlighted the critical role of their colleagues as facilitators of this process.

Peer and near-peer teaching has been effectively used in medical student education and has been shown to be as effective as faculty-led teaching.^16^ Less is known about the efficacy of peer teaching in graduate medical education; however this work suggests that residents perceive peer teaching as a primary source of team leadership training. Explicitly involving senior residents and other resuscitation team members in the development and implementation of a leadership curriculum may improve post-training transfer and dissemination by reinforcing training principles, reducing the exposure to contradictory information, and removing barriers to team leadership practice. It is also important to be aware that while peer teaching was cited as an effective means of leadership development, this educational resource generally lacks standardization. This may lead to inequity in accessibility and opportunities for acquiring leadership experience for underrepresented groups in medicine and, therefore, deserves additional attention.^17^

In addition to understanding clinical and curricular factors, it is important to understand the learners themselves. Our study revealed that residents draw on previous life experiences in developing their leadership skills. The leadership development literature has examined the impact of past experiences on leadership development. Residents may bring diverse prior leadership experiences to the trauma team leader role, and these experiences can strengthen a person’s belief in their own leadership abilities as well as influence leadership beliefs and practices. Incorporating previous leadership experiences into learning and on-the-job experiences may assist in leadership development.^18^ To be more effective, leadership training could acknowledge these residents’ previous experiences and build upon residents’ existing knowledge.

Residents acknowledged that the dual role of learner and leader had both advantages and disadvantages. Participants identified that support from the team was often very helpful in ensuring patient safety and their own success as a team leader. However, team involvement could at times be viewed as interference, undermining residents’ ability to lead, as well as their self-efficacy. In fact, many of the challenges identified by residents related to the team, rather than clinical care. Given that team leadership and team performance are interdependent, the emphasis on team members’ behaviors and relationships is not surprising. The importance of team followership is well recognized in the broader leadership and team science literature, but it has received comparatively little attention in healthcare.^19^ ^,^ ^20^ Exemplary followers proactively contribute to team goals and support the team leader in a positive way, whereas other followership styles may be less supportive.^21^

While not a focus of this work, gender-based differences in the team leadership experience were mentioned. Female residents reported facing more challenges in getting the team to recognize them as the team leader. This is consistent with prior qualitative work exploring the role of gender and team leadership during medical resuscitations in both EM and internal medicine.^22^ ^,^ ^23^ These findings highlight the importance of the team and team followership in promoting effective team leadership. Team leadership training programs could be supported through teamwork and team membership training for all members of the healthcare team, with a specific focus on eliminating disparate treatment of team leaders based on innate characteristics, such as gender.

In summary, this study identified individual and contextual factors that inform trauma leadership development among EM residents. An important consideration for leadership training is the impact of prior and current experiences on EM residents’ leadership development. Educators should also consider the institutional factors that may inhibit or contribute to the leadership development of EM residents.

## LIMITATIONS

This study had several limitations. First, the findings are from a small sample size at one urban, academic, Level 1 trauma center. Findings may not reflect the experiences of EM residents in other settings, as environmental factors (region, culture, resource availability) may have influenced responses. While qualitative studies aim to provide an in-depth understanding of specific contexts, future quantitative studies can expand on the aims of the current study to understand experiences of EM residents in other contexts. Selection bias is another potential limitation. Residents serving on non-EM rotations or who were off-site during the data collection period may have been less likely to participate. While the sample was balanced across men and women, only two participants (20%) reported a racial background other than White (Asian and multi-racial; native Pacific/Hawaiian Islander, White, and Asian descent)). However, this percentage is reflective of the average racial and ethnic makeup of EM residency classes over multiple years (2016–2022). Finally, reporting bias may have impacted the results. Coders had backgrounds in sociology, public health, and organizational psychology, but they did not have clinical expertise. The potential for bias was mitigated by having EM attending physicians review the findings throughout the coding process to provide context and interpret professional and institution-specific terminology.

## CONCLUSION

Emergency medicine residents learn about and develop leadership skills through multiple sources and experiences, many of which are outside the formal medical education curriculum. Additionally, various individual and team factors can support and/or inhibit leadership development. We encourage the development of leadership training programs that incorporate diverse training strategies that take into consideration EM residents’ prior leadership experiences and address some of the contextual factors that influence leadership development. More research is needed to identify the specific ways in which educators can leverage learners’ prior experiences and existing informal educational processes to develop more effective leadership training programs.

## Supplementary Information





