# Supplementary material for: Qualitative Study of Emergency Medicine Residents’ Perspectives of Trauma Leadership Development
Source: West J Emerg Med. 2023 Dec 20;25(1):122–8. doi: 10.5811/westjem.60098 (PMC10777183; doi:10.5811/westjem.60098)
Supplement: Supplementary file 1 [file wjem-25-122-s001.pdf]

Table S1: Domain Mapping of Interview Questions and Probes

Domain	Question or Probe
Introductory questions	Describe for me a typical trauma resuscitation.
	When you're the team leader, what do you do during a trauma resuscitation?
Source of leadership skills	Where have you learned resuscitation team leadership skills? This can be within or outside of medicine, formal or informal.
	What was useful about this experience/training/resource?
Implementation of newly acquired leadership skills: barriers and facilitators	What do you do well when leading a resuscitation?
	What goes into your decision of whether to try out a new leadership skill during a resuscitation? This could include individual, team, unit, or system-level factors.
	What's challenging for you when leading a resuscitation?
	What makes implementing a new skill more difficult?
	You mentioned X as a barrier; can you think of any other barriers you or your peers might have faced?<br><br>What would you change?
Formal resuscitation/code leadership training: content, characteristics of the training and delivery	Tell me about any formal code leadership training you've received.
	How was the transfer to the clinical setting?<br><br>What about the training or its delivery helped you implement the skills? What wasn't useful?
	If you were to design resuscitation team leadership training, what would it look like?
Dissemination of team leadership skills: does it happen, how, and by whom	How do you see team leadership skills and information being passed on?
	What is your role in disseminating (learned) trained leadership skills to your peers?
	What's a senior resident's role with respect to junior residents?
Closing free response	Is there anything else you would like to share about your experience with learning, applying, or disseminating team leadership skills?
