# Supplementary material for: Qualitative Study of Emergency Medicine Residents’ Perspectives of Trauma Leadership Development
Source: West J Emerg Med. 2023 Dec 20;25(1):122–8. doi: 10.5811/westjem.60098 (PMC10777183; doi:10.5811/westjem.60098)
Supplement: Supplementary file 2 [file wjem-25-122-s002.pdf]

**Table S2: Central Themes and Exemplar Quotes**

| Themes | Representative Quotations |
|---|---|
| **Sources of Leadership Development** | |
| ***Sub-Themes*** | |
| Life Experiences | "For me I used to play baseball, I was in [youth organization] and all the like typical young white boy things, and that has made me do it before, so I guess I come into it with some level of like I've had to be responsible for other people before. […] After that you kind of develop the confidence in yourself, but still are open to suggestion and I think that's pretty much been one of the big drivers for me." (Participant 7) |
| | "So in college I played sports, and I was also like the president of our newspaper, so I like did the roles, but it wasn't like formal training. It was just kind of like you step in and you kind of feel your way through things, which I think is a lot more like what we do here is you kind of just figure out how it goes. And I think that's why in a way, rugby was helpful because it was a sport that I didn't know how to play that I kind of just wandered onto the field and somebody taught me how to do it along the way." (Participant 8) |
| Observations | "How would I … part of it is in intern year you see it and that's a part of your just kind of unnamed curriculum where you just learn though osmosis by seeing things. […] Seeing the chaos of a full trauma is definitely necessary." (Participant 7) |
| | "I feel like a lot of intern year there's definitely learning medicine, but it's a lot of observing how people do things in the next level, and it's just like I'm kind of watching people for a whole year and just seeing how they do it. […] but that role very much disappears once you start … once you start doing it. Then all you really can do is look to the attendings who very often are not … you don't get to see them do it either, so …" (Participant 10) |

| | |
|---|---|
| | "So we've gotten … so first of all I watched trauma docs do it all last year and I worked many traumas just as an intern, so I just observed the way it happens. So that's probably honestly like the bulk of my experience so far is watching other people do it in real time and sort of seeing who does it well, what can go better, some of the problems people face and kind of taking that all into account." (Participant 3) |
| Low-Stakes Supervised Clinical Practice | "When I was an intern, and what I'll do with interns sometimes, especially emergency interns because they're the ones that are going to end up being in the trauma doc role, is after they've been working for a while in modified trauma sometimes, or just patients who are sick enough to like be on a backboard, still have them basically run through the full kind of script of doing a trauma resuscitation, even though they're not that sick of a patient, just to kind of practice. And then once interns have been through a couple months of ER rotations, then you will just have them run modifieds because there's less people in the room, but they're still patients that need resuscitation and gives you a little bit of a chance to practice before you get thrown into a full trauma." (Participant 11) |
| | "I had a couple of opportunities as an intern. I had support of the trauma doc, and they would say, Hey, this is a lower-level trauma. Do you want to practice running through this algorithm, or practice being the trauma doc? And that made me very nervous, but I sort of agreed, begrudgingly, to do it because I knew that it would make actually stepping into the trauma doc role a little less nerve wracking." (Participant 10) |
| **Dual Leader- Learner Role** | |
| ***Sub-Themes*** | |
| Leader Role | "As the trauma doc, we're kind of the central figure. So there's a lot of times they [general surgery services] provide input and then I will kind of direct somebody to do it, or do it myself, but we are kind of supposed to |

| | |
|---|---|
| | be the hub and then there's various spokes of providing input in their own expertise." (Participant 5) |
| | "The expectation is that I know the trauma algorithm, which I do, that I run through it and that I try to stand at the head of the bed and be loud and command the room to the best of my abilities with the expectation being that like I am not going to be as skilled at that as some of my older, more seasoned colleagues. I'd like to say that that would be the expectations of the trauma team, too." (Participant 3) |
| Learner Role | "It's not necessarily my job to know every answer because I have people that are much more experienced to whisper in my ear about that stuff, but it is my job to like verbalize what's happening and sort of like manage the situation.´ (Participant 3) |
| | "I think ultimately the trauma doc role is like an opportunity for you to have this like leadership and like practice running a resuscitation, but it's sort of the initial resuscitation in terms of like assessing this initial, primary survey, doing the ABCs …" (Participant 2) |
| | "I'm still at the point, because I'm so early on, where I'm like questioning if I even know what the right thing is to do next in terms of the medicine, and so I second-guess myself and it's hard to feel confident if you don't actually know what you think is the right move medically. And then the confidence comes across and like I think there's … you can tell when the trauma code leader knows what they're doing. And I don't know if I'm quite at that point yet." (Participant 3) |
| | "Yeah, I guess part of it is this role is … I feel the role is kind of hard because you have to … I don't know … you have to have the confidence in something that you haven't really done before, because if you're not confident, then everyone will just take it away from |

| | |
|---|---|
| | you here and start speaking over you and you're kind of flushed under the rug.” (Participant 7) |
| **Contextual Factors** | |
| ***Sub-Themes*** | |
| Professional Hierarchy | |
| | “…It’s like an unspoken hierarchy that exists in medicine and especially exists in a situation like that, so people tend to listen to whoever they know is the more senior person at the bedside.” (Participant 9) |
| | “I think there are times as like a junior resident where you just kind of get pushed where the waves take you. And if attendings and your surgery consultants, and all these other people are saying these different things during resuscitation you just say, okay, and it just devolves into this reactionary thing where we're like doing a bunch of random things as opposed to a nice flow where we know exactly, we're going to do this first…” (Participant 4) |
| Gender | “I think especially from my experience maybe as a young lady in the emergency department that sometimes it can be difficult to … or I got a lot of feedback in the past about deepening my voice, or speaking more loudly, or asserting my dominance.” (Participant 1) |
| | “I think, interestingly, I was asked by some of our junior female residents to try to organize a talk on like specifically running resuscitations, trauma resuscitations, being a female provider. And historically, I've been coached by other people that like there's a little bit of a mountain to overcome in just how you're perceived in that role.” (Participant 11) |
| Physical Environment | “The room is unbelievably packed. I'm standing now behind the anesthesiologist at the head of the bed, trying to squeeze my way in, yelling at everybody so that they pay attention. … And so I get to stand behind like two people, yelling at everybody. And the nurse |

| | |
|---|---|
| | [Inaudible] and I just kind laugh about it sometimes." (Participant 7) |
| | "I think that the … so what we were most recently saying, the physical issues of space make it so that maybe I can't do my primary survey as quickly as I can" (Participant 8) |
